# A nomogram to predict sarcopenia in middle-aged and older women: a nationally representative survey in China

**DOI:** 10.3389/fpubh.2025.1410895

**Published:** 2025-02-05

**Authors:** Jiayi Yang, Zihao Chen, Xinxin Dai, Liyao Jiang, Liyan Dai, Yu Zhao

**Affiliations:** ^1^Department of Gynecology and Obstetrics, The Second Affiliated Hospital of Wenzhou Medical University, Wenzhou, Zhejiang, China; ^2^Department of Orthopaedic Surgery, The Second Affiliated Hospital and Yuying Children Hospital of Wenzhou Medical University, Wenzhou, China; ^3^Department of Gynecology and Obstetrics, The First Affiliated Hospital of Wenzhou Medical University, Wenzhou, Zhejiang, China; ^4^Department of Gynecology and Obstetrics, Lucheng District People's Hospital, Wenzhou, Zhejiang, China

**Keywords:** women's health, middle and aged women, sarcopenia, nomogram, risk factors

## Abstract

**Background:**

Sarcopenia is a disease characterized by losing muscle mass, strength, and function with age. Studies have shown that sarcopenia is generally higher in women than in men. Therefore, this study used the 2015 China Health and Retirement Longitudinal Study (CHARLS) data to explore further the risk factors associated with sarcopenia in middle-aged and older Chinese women.

**Methods:**

In this study, data from the 2015 CHARLS database were analyzed, comprising 7,805 eligible participants. Participants were categorized into either the sarcopenia group (*n* = 2,160) or the non-sarcopenia group (*n* = 5,645) based on the presence or absence of sarcopenia. Through the utilization of logistic regression analysis, multiple risk factors were identified. Additionally, the predictive value of these risk factors was assessed by applying receiver operating characteristic (ROC) curve analysis. Subsequently, a visual nomogram prediction model was developed by incorporating the identified risk factors into R4.1.2 software.

**Results:**

Age, area, education, marriage, waist circumference, stroke, body pain, depression, and region may be closely related to Chinese women with sarcopenia. In addition, this study integrated these sarcopenia-related variables into a comprehensive index, and ROC analysis results showed that the AUC of the composite index was 0.738.

**Conclusions:**

This study found that sarcopenia in Chinese women may be closely related to age, waist, education, marriage, area, stroke, physical pain, depression, and region. In addition, this study constructs a nomogram to help clinicians better screen potential female patients with sarcopenia.

## Introduction

Sarcopenia is characterized by the loss of muscle mass, strength, and function that occurs with aging (1). It is a natural part of the aging process, and after middle age, muscle mass typically declines by about 1% per year (2). The consequences of sarcopenia can be significant, including decreased mobility, increased risk of falls and fractures, and decreased quality of life. Moreover, it can also lead to an increased risk of chronic diseases such as diabetes, obesity, and osteoporosis (3–5). Several factors contribute to the development of sarcopenia, including hormonal changes, decreased physical activity, poor nutrition, and inflammation (3).

Although sarcopenia is common in men and women, Petermann-Rocha et al. found that the prevalence of sarcopenia was higher in women than in men (6). Women generally exhibit lower muscle mass than men, starting from a younger age (7). Consequently, they are at an elevated risk for developing sarcopenia as they age. Hormonal changes during menopause, such as a decrease in estrogen, can further accelerate muscle loss and increase the risk of sarcopenia in women (8). Additionally, certain lifestyle factors may impact sarcopenia risk differently in women. For example, women are more likely to engage in low-impact exercises like walking or yoga, which may not provide enough resistance training to maintain muscle mass. High-impact exercises, such as weightlifting or resistance training, are more effective in preserving muscle mass (9). Hence, it is crucial to prioritize research exploring the connection between sarcopenia and middle-aged and older women.

The China Health and Retirement Longitudinal Study (CHARLS) is a comprehensive research project to gather high-quality microdata representing individuals and families aged 45 and above in China (10). Its primary purpose is to analyze the population aging problem in China and promote interdisciplinary research on this issue. The initial phase of CHARLS, known as the national baseline survey, took place in 2011. It encompassed 150 county- and 450 village-level units and involved ~17,000 people from around 10,000 households. To ensure the continuity of the study, subsequent surveys will be conducted every 2–3 years, allowing for longitudinal tracking of the participants. The ongoing CHARLS project is valuable, providing comprehensive and periodically updated data on aging and health trends among middle-aged and older individuals in China.

This study used the 2015 CHARLS data to explore further the risk factors associated with sarcopenia in middle-aged and older Chinese women. By analyzing survey data from a large sample, we sought to reveal the prevalence of sarcopenia in middle-aged and older women and possible risk factors in order to provide a scientific basis for prevention and treatment.

## Methods

### Study design

**Figure 1:**
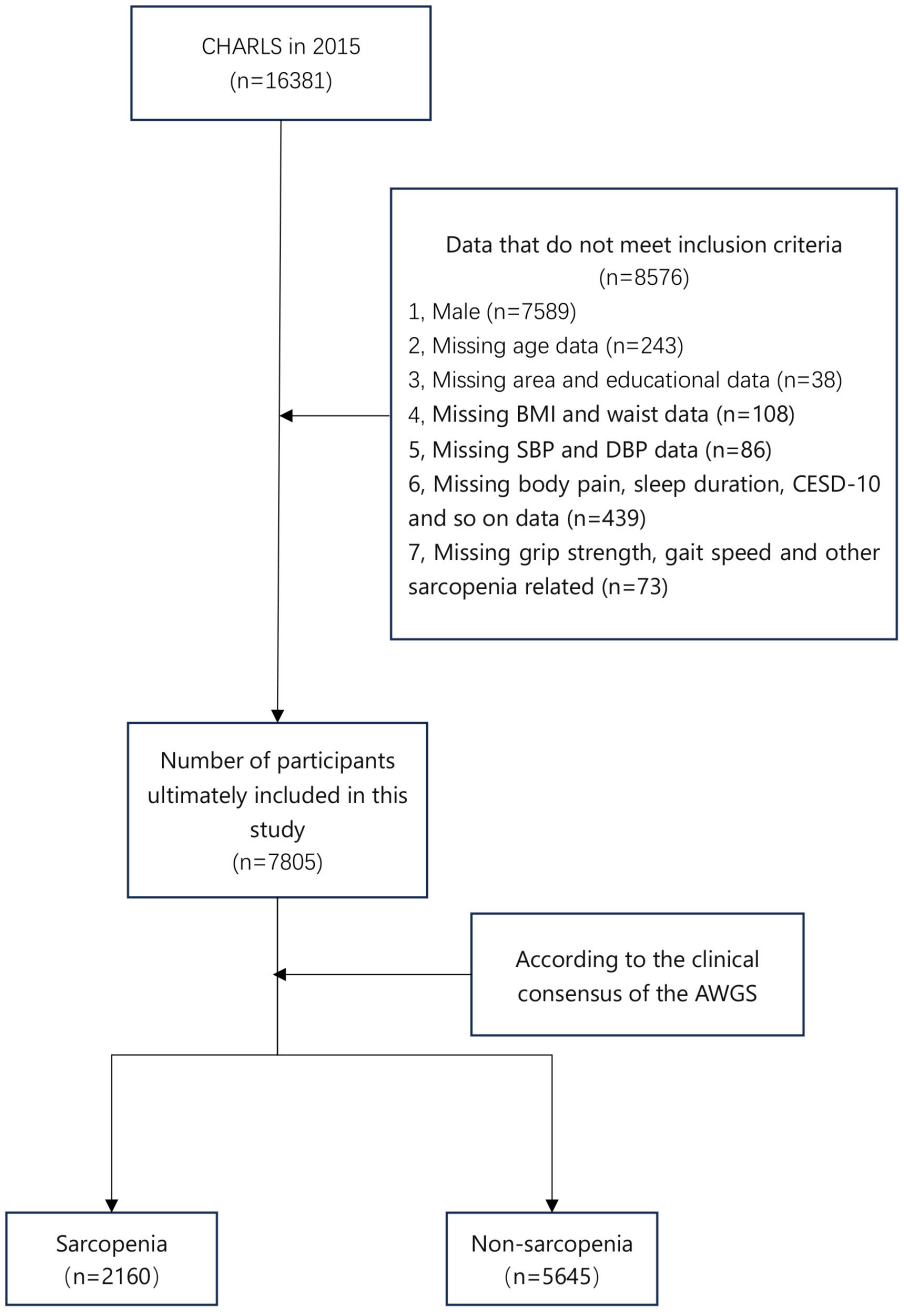
Flow chart of participants in the study. CHARLS, China Health and Retirement Longitudinal Study; BMI, body mass index; SBP, systolic blood pressure; DBP, diastolic blood pressure; CESD-10, 10-item Center for Epidemiological Studies Depression Scale; AWGS, Asian Working Group for Sarcopenia.

The study included 7,805 eligible participants from the 2015 CHARLS database. According to the presence or absence of sarcopenia, the patients were divided into the sarcopenia group (*n* = 2,160) and non-sarcopenia group (*n* = 5,645) (Figure 1). Age, waist, body mass index (BMI), sleep duration in the past month, education, smoking history, region, region, marriage, depression, high blood pressure, dyslipidemia, diabetes or high blood sugar, chronic lung disease, heart disease, stroke, digestive disease, emotional problems, memory-related diseases, arthritis or rheumatism, asthma, frequency of drinking in the past year, body pain, liver disease, and cancer were included in the study.

In addition, this study divides the study population into seven regions based on geographical, economic, and cultural considerations. Northeast (Heilongjiang, Jilin, and Liaoning provinces), East (Anhui, Fujian, Jiangsu, Jiangxi, Zhejiang, Shandong, and Shanghai), North (Hebei, Shanxi, Inner Mongolia Autonomous Region, Tianjin, and Beijing), Central (Hubei, Hunan and Henan provinces), South (Guangdong and Guangxi provinces), Southwest (Yunnan, Guizhou, Sichuan and Chongqing) and Northwest China (Qinghai, Shaanxi, Gansu and Xinjiang Autonomous Region). The survey did not include Hainan Province, Taiwan Province, Ningxia Autonomous Region, Tibet Autonomous Region, Hong Kong Special Administrative Region, and Macao Special Administrative Region.

Self-reports were used to determine whether participants had hypertension, dyslipidemia, diabetes or high blood sugar, cancer, chronic lung disease, liver disease, heart disease, stroke, kidney disease, digestive disease, emotional problems, arthritis or rheumatism, memory-related diseases, and asthma. For example, participants were asked, “Has a doctor ever told you that you have dyslipidemia?” If the participant answered “Yes” to this question, the participant was considered to have dyslipidemia. In addition, according to Chinese clinical guidelines, participants are also considered to have high blood pressure when their average systolic blood pressure (SBP) is greater than or equal to 140 mmHg or their average diastolic blood pressure (DBP) is greater than or equal to 90 mmHg.

Depression was evaluated using the 10-item Center for Epidemiological Studies Depression Scale (11) (CESD-10) in the CHARLS questionnaire. Participants with a CESD-10 score of 10 or higher were categorized as having depressive symptoms.

### The definition of sarcopenia

According to the consensus of the 2019 version of the Asian Working Group for Sarcopenia (AWGS) (12), sarcopenia is defined as “age-related loss of muscle mass coupled with low muscle strength and/or low physical performance.” Low muscle strength is primarily defined as a grip strength greater than 28 kg for men and less than 18 kg for women. Low physical performance was defined as a 6-meter walk of less than 1.0 m/s or five chair standing tests greater than or equal to 12 s.

This study divided participants into sarcopenia and non-sarcopenia groups based on whether they met diagnostic criteria for low muscular strength or low physical performance.

### Inclusion and exclusion criteria

The inclusion criteria were: (1) Female, (2) The diagnostic criteria of sarcopenia conform to the consensus of the 2019 version of the AWGS. The exclusion criteria were: (1) Clinical baseline characteristics were missing, such as education, BMI, etc. (2) Male.

### Statistics

Data distribution was assessed using the Shapiro-Wilk test. Patient characteristics were described using median (interquartile range [IQR]) or frequency and percentage, as appropriate. A non-parametric test (Mann-Whitney *U*-test or Kruskal-Wallis test) was employed for data with non-normal distribution or heterogeneity of variances. Categorical variables were presented as percentages and analyzed using the Pearson Chi-squared test. Relevant risk factors (*P* < 0.05) were identified through multivariate logistic regression analysis and integrated into a composite index. The predictive performance of this composite index was evaluated using the receiver operating characteristic (ROC) curve. All statistical analyses were conducted using SPSS software (version 26.0; SPSS et al., USA).

Furthermore, the final risk factors were integrated into the R4.1.2 software (R Foundation for Statistical Computing, Vienna, Austria) to establish a nomogram prediction model. The effectiveness of the model's predictions was assessed using the consistency index (C-index), with a range of 0.5–1.0. Accuracy was positively associated with the C-index value. The calibration curve, which included an image comparison of predicted and actual risks, was used to evaluate the prediction consistency. The conformity of the model was determined by how closely the predicted risk aligned with the standard curve.

## Results

A total of 7,805 female participants were included in the study, of whom 2,160 had sarcopenia, and 5,645 did not. There were significant differences in age, waist, BMI, sleep duration over the past month, education, smoking history, region, area, marriage, depression, hypertension, dyslipidemia, diabetes or high blood sugar, chronic lung disease, heart disease, stroke, digestive diseases, emotional problems, memory-related diseases, arthritis or rheumatism, asthma, and body pain (all *P* values < 0.05). There were no significant differences in the frequency of drinking in the past year, liver disease, and cancer (Table 1).

**Table 1:** Comparison of clinical baseline features between the two groups.

**Variables**	**Non-sarcopenia (*n* = 5,645)**	**Sarcopenia (*n* = 2,160)**	***P* value**
Age (years)	58 (52–65)	66 (59–74)	< 0.001
Waist (cm)	86.0 (79.0–92.6)	86.6 (79.0–94.6)	0.003
BMI (kg/m^2^)	24.20 (21.93–26.61)	23.71 (21.13–26.57)	< 0.001
Sleep duration over the past month (hours)	6.0 (5.0–8.0)	6.0 (4.5–8.0)	< 0.001
**Education**, ***n*** **(%)**			< 0.001
No formal education	4672 (82.8)	1902 (88.1)	
Elementary school	276 (11.3)	184 (8.5)	
Middle school	193 (3.4)	46 (2.1)	
High school or higher	232 (4.1)	28 (1.3)	
**Smoking history**, ***n*** **(%)**			< 0.001
Never smoked	5,232 (92.6)	1,894 (87.7)	
Have smoked	419 (7.4)	267 (12.3)	
**Frequency of drinking in the past year**, ***n*** **(%)**			0.069
No drinking	4,762 (84.3)	1,863 (86.1)	
Drink but less than once a month	423 (7.5)	120 (5.5)	
Drink more than once a month	467 (8.3)	181 (8.4)	
**Region**, ***n*** **(%)**			< 0.001
Northeast	431 (7.6)	183 (8.5)	
East	1,840 (32.6)	660 (30.6)	
North	634 (11.2)	317 (14.7)	
Central	915 (16.2)	273 (12.6)	
South	494 (8.8)	139 (6.4)	
Southwest	966 (17.1)	352 (16.3)	
Northwest	365 (6.5)	236 (10.9)	
**Area**, ***n*** **(%)**			< 0.001
Urban	1,372 (24.3)	397 (18.4)	
Rural	4,273 (75.7)	1,763 (81.6)	
**Marriage**, ***n*** **(%)**			< 0.001
Unmarried	611 (10.8)	542 (25.1)	
Married	5,034 (89.2)	1,618 (74.9)	
Depression, *n* (%)	2,415 (42.8)	1,095 (50.7)	< 0.001
Hypertension, *n* (%)	2,067 (36.6)	1,066 (49.4)	< 0.001
Dyslipidemia, *n* (%)	498 (8.8)	233 (10.8)	0.008
Diabetes or high blood sugar, *n* (%)	298 (5.3)	167 (7.7)	< 0.001
Cancer, *n* (%)	71 (1.3)	35 (1.6)	0.217
Chronic lung diseases, *n* (%)	408 (7.2)	251 (11.6)	< 0.001
Liver disease, *n* (%)	203 (3.6)	83 (3.8)	0.607
Heart disease, *n* (%)	614 (10.9)	365 (16.9)	< 0.001
Stroke, *n* (%)	68 (1.2)	66 (3.1)	< 0.001
Kidney disease, *n* (%)	303 (5.4)	151 (7.0)	0.006
Digestive disease, *n* (%)	1,322 (23.4)	618 (28.6)	< 0.001
Emotional problems, *n* (%)	65 (1.2)	43 (2.0)	0.005
Memory-related disease, *n* (%)	51 (0.9)	39 (1.8)	0.001
Arthritis or rheumatism, *n* (%)	1,883 (33.3)	949 (43.9)	< 0.001
Asthma, *n* (%)	140 (2.5)	100 (4.6)	< 0.001
Body pain, *n* (%)	1,834 (32.5)	1,023 (47.4)	< 0.001

BMI, body mass index.

A binary multi-factor logistics regression analysis was used to analyze the above-related variables, and it was finally found that age, area, education, marriage, waist, stroke, body pain, depression, and region were risk factors for female sarcopenia (*P* values were all < 0.05) (Table 2). In addition, these risk factors were integrated into a composite index (age + area + education + marriage + waist + stroke + body pain + depression + region).

**Table 2:** Logistic regression analysis of female sarcopenia related factors.

**Variables**	***P***	**OR**	**95% CI**
Age (years)	< 0.001	1.079	1.072–1.086
Area	< 0.001	1.514	1.317–1.740
Education	0.039	0.903	0.820–0.995
Marriage	< 0.001	0.759	0.654–0.881
Waist	0.026	1.005	1.001–1.009
Stroke	0.002	1.773	1.225–2.566
Body pain	< 0.001	1.634	1.4456–1.834
Depression	0.040	1.127	1.006–1.262
Region	< 0.001		
East	0.001	0.651	0.502–0.845
North	< 0.001	0.472	0.384–0.579
Central	0.015	0.752	0.597–0.947
South	< 0.001	0.407	0.323–0.513
Southwest	< 0.001	0.338	0.258–0.444
Northwest	< 0.001	0.447	0.357–0.559

**Figure 2:**
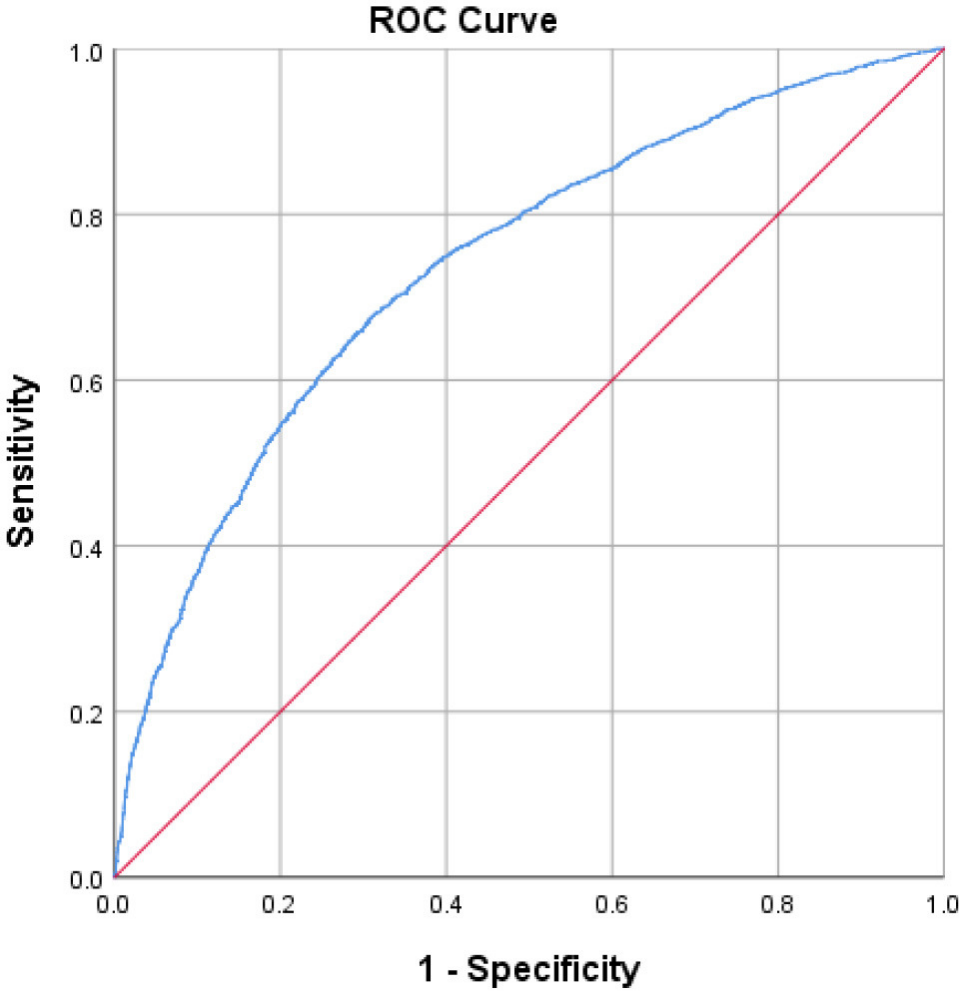
Discriminatory accuracy for predicting female sarcopenia by receiver operator characteristics (ROC) analysis calculating area under the curve (AUC).

**Figure 3:**
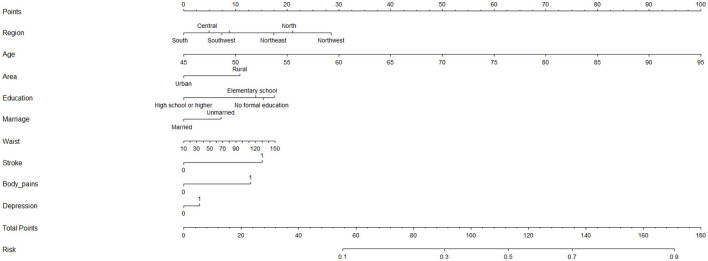
A nomogram was established to predict the female sarcopenia. Identify the individual characteristics: For each characteristic (e.g., Region, Age, Area, etc.), locate the corresponding value for the individual. Draw a vertical line from the value to the “Points” scale at the top to determine the score for that characteristic. Calculate the total score: Add up all the points from the individual characteristics to calculate the “Total Points.” Estimate the risk: Match the total score to the “Risk” scale at the bottom of the chart to obtain the estimated probability of the risk. Variable Descriptions: Region: The geographic region where the individual resides (South, Southwest, Northeast, Northwest, or Central). Age: The individual's age, ranging from 45 to 95 years. Area: Indicates whether the individual lives in an urban or rural area. Education: The highest level of education attained: High school or higher; Elementary school; No formal education; Marriage: Whether the individual is married or unmarried. Waist: Waist circumference in centimeters, ranging from 10 to 150. Stroke: Indicates whether the individual has a history of stroke (0 = No, 1 = Yes). Body_pains: Indicates whether the individual experiences body pains (0 = No, 1 = Yes). Depression: Indicates whether the individual has symptoms of depression (0 = No, 1 = Yes). Risk Scale: The risk scale at the bottom translates the total score into a probability value (ranging from 0.1 to 0.9). This value represents the likelihood of the outcome being assessed (e.g., a health condition or disease).

**Figure 4:**
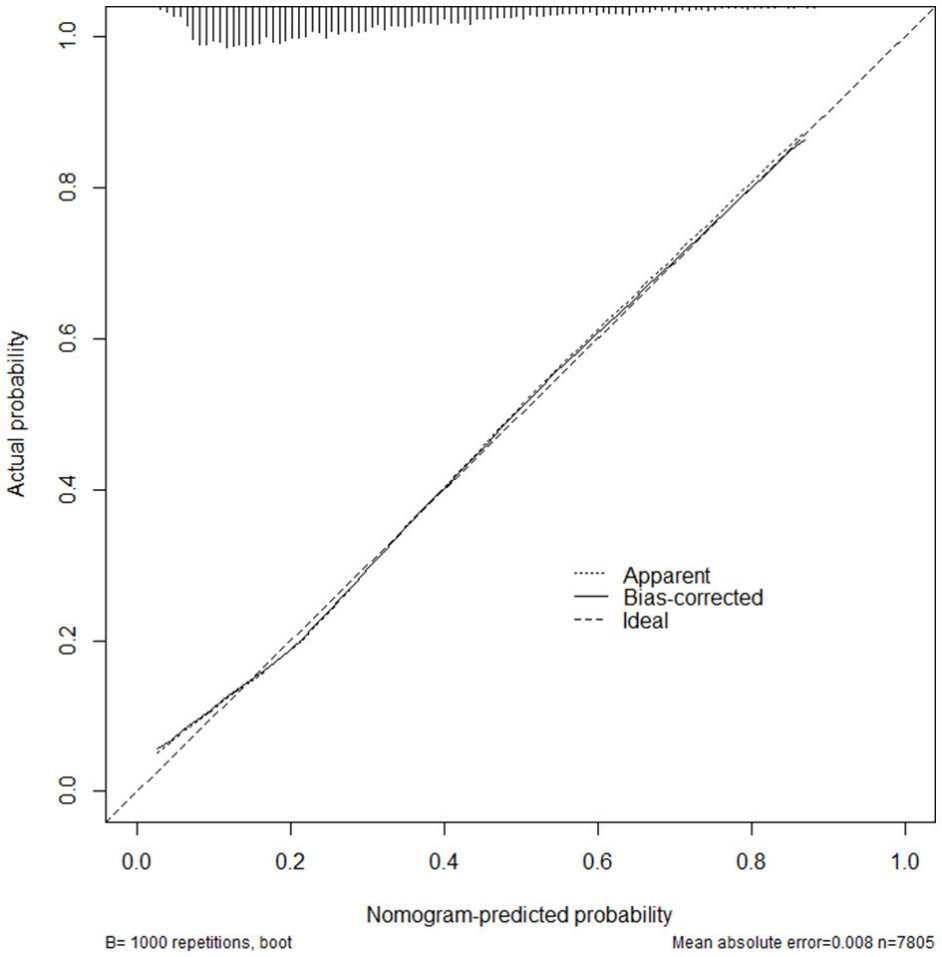
Calibration curve for nomogram prediction of female sarcopenia.

ROC curve analysis showed that the area under the curve (AUC) of the composite indicator for predicting sarcopenia was 0.738 (95% CI 0.725–0.750 *p* < 0.001) (Figure 2). In addition, this study established a nomogram to visually screen Chinese female patients with sarcopenia by screening relevant risk factors (Figure 3). After 1,000 repetitions of bootstrap self-sampling, the C-index of the model is 0.738, indicating that the agreement between the predicted value and the actual observed value meets the standard. Moreover, Figure 4 shows that the calibration curve is well-fitted.

## Discussion

A series of variables, such as age, BMI, and educational background, were included in this study. It was finally found through logistics regression analysis and other related analysis methods that age, area, education background, marriage, waist, stroke, body pain, depression, and region may be closely related to female sarcopenia patients. In this study, these sarcopenia-related variables were integrated into a composite indicator, and ROC analysis was used to evaluate the efficacy of this composite indicator in predicting female sarcopenia patients. The results of the ROC analysis showed that the AUC of this composite index was 0.738, which was effective in evaluating sarcopenia. In addition, this study created a nomogram of the risks associated with sarcopenia to help clinicians better screen women with sarcopenia.

Long-term living environment is also closely related to the prevalence of sarcopenia. In this study, similar to previous studies, the prevalence of sarcopenia was generally higher in rural areas than in urban areas (13). The prevalence of sarcopenia is generally higher in rural areas, possibly due to a series of reasons such as poor environment and lower education level in rural areas. In China, seven regions are divided according to economic and geographical characteristics, including Northeast, North, East, Central, South, Southwest, and Northwest. Various factors, including environmental factors, genetic background, and medical resources, may influence the differences in the prevalence of sarcopenia between different regions. In this study, sarcopenia patients were mainly found in southern China. As the most economically active region in China, the southern region has attracted many people to migrate and settle here. Sarcopenia is a genetic disorder that is passed on through genetic mutations. Due to the large population in the southern region, the susceptibility to genetic mutations is relatively high, which may partly explain the results of this study.

Numerous risk factors were linked to sarcopenia in recent research. Zhang et al. found that imbalances in protein metabolism (protein degradation over protein synthesis), which lead to severe reductions in muscle strength and motor capacity, are associated with regulation of the ubiquitin-proteasome system, oxidation reactions, and autophagy, as well as potential novel mechanisms, including altered miRNA profiles and gut microbiota (14). At the same time, sarcopenia is closely related to glucose metabolism, and studies have found that sarcopenia is related to hypoglycemia treatment in skeletal muscle, Inflammation, insulin resistance, and impaired intramuscular blood flow regulation significantly affect how skeletal muscles process glucose (15). Aging, type 2 diabetes, and obesity are all associated with changes in the metabolism of fatty acids (FAs); lipid buildup inside muscle cells is a major cause of muscle insulin resistance and ceramide formation. One of the main indicators of sarcopenia is muscular fat infiltration (16). In addition, gynecological cancer patients are at risk of sarcopenia, Cancer-related malnutrition has a complicated etiology that includes metabolic abnormalities, such as lipolysis and proteolysis, and a systemic pro-inflammatory state of malignancy (17, 18).

The relationship between age and sarcopenia is a complex one. Sarcopenia is commonly associated with aging and is often seen in older adults (19). As we age, several physiological changes contribute to the development of sarcopenia. These include hormonal changes, decreased physical activity, inadequate nutrition, and chronic inflammation (20, 21). These factors can lead to a progressive loss of muscle mass, strength, and function. Similar to the above study, age is also an essential factor in female sarcopenia patients in this study.

While age is a significant risk factor for developing sarcopenia, it is not the sole determinant. Lifestyle factors such as physical activity level, dietary habits, and overall health also play a role (22). This study found that female sarcopenia patients were generally less educated and unmarried. Highly educated people and married people tend to devote their spare time to exercise and nutrition intake to maintain a healthy state, which prevents the occurrence of sarcopenia to a certain extent (23, 24). In addition, similar to the findings of Sousa-Santos et al. (25), this study found that marriage and high education were negatively correlated with the occurrence of sarcopenia.

In this study, the waist of female patients with sarcopenia was significantly higher than that of non-sarcopenia patients. Waist circumference is frequently utilized as an indicator of abdominal obesity. There is growing evidence of a link between sarcopenia and abdominal obesity (26, 27). The findings of Kim et al. (28) also support the conclusion of this study that obesity is significantly related to the occurrence of sarcopenia. There are a few mechanisms through which abdominal obesity may contribute to sarcopenia. Chronic inflammation and insulin resistance, often in people with abdominal obesity, can negatively affect muscle protein synthesis and promote muscle breakdown (26). Additionally, adipose tissue produces various substances called adipokines that can harm muscle function and metabolism (29).

As an aging, degenerative disease, sarcopenia is closely related to many chronic diseases, such as depression, stroke, and body pain. People with depression may experience reduced physical activity, lack of motivation, and interest, which can lead to a loss of muscle mass. In addition, because people with depression may suffer from distress and distress, they may neglect or abandon proper eating and exercise habits in terms of self-care, which can also lead to further declines in muscle function and mass (30, 31). Because strokes damage the neural pathways between the brain and muscles, patients can experience muscle atrophy, decreased muscle strength, and dysfunction after a stroke. In addition, patients often require a long rehabilitation and recovery process after a stroke, which can lead to chronic inactivity and loss of physical function, further exacerbating the extent of sarcopenia (32, 33). There are some correlations between body pain and sarcopenia. Although body pain is not a direct symptom of sarcopenia, there can be an interaction between the two. Individuals with sarcopenia often encounter a decline in muscle mass and strength, which can result in abnormal loads and imbalances in the body. Consequently, this can provoke muscle and joint pain. However, pain can lead to movement restrictions that prevent the muscles from being adequately stimulated and used. This lack of movement and activity may promote muscle atrophy and decreased function, worsening sarcopenia (34, 35).

However, there are several limitations to this study. First of all, this study is a cross-sectional study, so there are difficult to control confounding factors, recall bias, and other limitations. Second, due to the significant absence of certain sarcopenia-related variables, such as participants' daily activity levels, this study did not include them. Finally, due to the absence of some biomarker data in the 2018 CHARLS data, the 2015 CHARLS data were included in this study. These studies may lead to some bias in the results of this study.

## Conclusion

This study found that sarcopenia in Chinese women may be closely related to age, waist, education, marriage, area, stroke, physical pain, depression, and region. In addition, this study constructs a nomogram to help clinicians better screen potential female patients with sarcopenia.

## Data Availability

The datasets presented in this study can be found in online repositories. The names of the repository/repositories and accession number(s) can be found in the article/supplementary material.
